# Case report: reinitiating pembrolizumab treatment after small bowel perforation

**DOI:** 10.1186/s12885-019-5577-5

**Published:** 2019-04-24

**Authors:** Tim N. Beck, Alexander E. Kudinov, Essel Dulaimi, Yanis Boumber

**Affiliations:** 1 Molecular and Cell Biology and Genetics, Drexel University College of Medicine, Philadelphia, PA 19129 USA; 2 Program in Molecular Therapeutics, Fox Chase Cancer Center, Philadelphia, PA 19111 USA; 3 Department of General Surgery, Cleveland Clinic, Cleveland, OH 44195 USA; 4 Department of Internal Medicine, University of New Mexico, Albuquerque, NM 87131 USA; 5 Department of Pathology, Fox Chase Cancer Center, Philadelphia, PA 19111 USA; 6 Department of Hematology/Oncology, Fox Chase Cancer Center, Philadelphia, PA 19111 USA; 7 Institute of Fundamental Medicine and Biology, Kazan Federal University, Kazan, Russia

**Keywords:** Pembrolizumab, Bowel perforation, Immunotherapy, Immune-related adverse events, Toxicity, Cancer, PD-1, CTLA4, PD-L1, Immune checkpoint inhibitors

## Abstract

**Background:**

Immune checkpoint inhibitors (ICIs) have emerged as paradigm shifting treatment options for a number of cancers. Six antibodies targeting the immune checkpoint proteins programmed cell death 1 (PD-1), programmed cell death 1 ligand 1 (PD-L1) or cytotoxic T-lymphocyte associated protein 4 (CTLA4) have been approved. In some cases, response rates have been impressive, but not uniformly so and not consistently; similarly, toxicity to this class of therapeutic is often unpredictable and can be life threatening. Predicting treatment response and toxicity are two main obstacles to truly individualize treatment with ICIs. One of the most severe and life-threatening adverse events is colitis induced colonic perforation, estimated to occur in 1.0 to 1.5% of patients treated with ICIs. An important question to address is, under what circumstances is it appropriate to reinitiate ICI treatment post-bowel perforation?

**Case presentation:**

The patient is a 62-year-old woman, who presented with stage IV lung cancer. Immunohistochemical staining indicated that 80% of the patient’s tumor cells expressed PD-L1. The patient was started on a three-week cycle of pembrolizumab. Subsequent reducing in tumor burden was observed within ten weeks. Initially, pembrolizumab was tolerated fairly well, with the exception of immunotherapy related hypothyroidism. However, the patient experienced a second, more serious immune-related adverse event (irAE), in the form of enteritis, which led to small bowel perforation and necessitated exploratory laparotomy.

The concerning part of the small bowel was resected, and a primary anastomosis was created. Based on the pathological and surgical findings, the patient was diagnosed with pembrolizumab-associated small bowel perforation. The patient recovered well from surgery and, considering the patient’s remarkable response to treatment, a collective decision was made to reinitiate pembrolizumab on post-operative day twenty-eight. The patient is continuing her immunotherapy with ongoing partial response and is able to continue her full-time job.

**Conclusions:**

This case report highlights the challenges of identifying patients likely to respond to ICIs and those that are likely to experience irAEs and it discusses the impressive work that has been done to start to address these challenges. Lastly, the topic of reinitiating pembrolizumab treatment even after colonic perforation is discussed.

**Electronic supplementary material:**

The online version of this article (10.1186/s12885-019-5577-5) contains supplementary material, which is available to authorized users.

## Background

Immunotherapy has emerged as a promising treatment approach for several different types of cancer, including malignant melanoma [1], mismatch repair deficient colorectal cancer [2], non-small cell lung cancer (NSCLC) [3–5] and others. Programmed cell death-1 (PD-1), cytotoxic T-lymphocyte associated protein 4 (CTLA4) and programmed cell death ligand-1 (PD-L1) are three molecular targets of particular focus. Blocking PD-1/PD-L1 or CTLA4 activity prevents initiation of immune-inhibitory signals within activated T-cells and thereby sustaining the immune response [6]. Two major challenges surrounding this class of inhibitors are being actively investigated to further personalize treatment: 1) identify molecular markers predictive of treatment response rates and 2) identify molecular markers predictive of immune-related adverse events (irAEs).

**Fig. 1:**
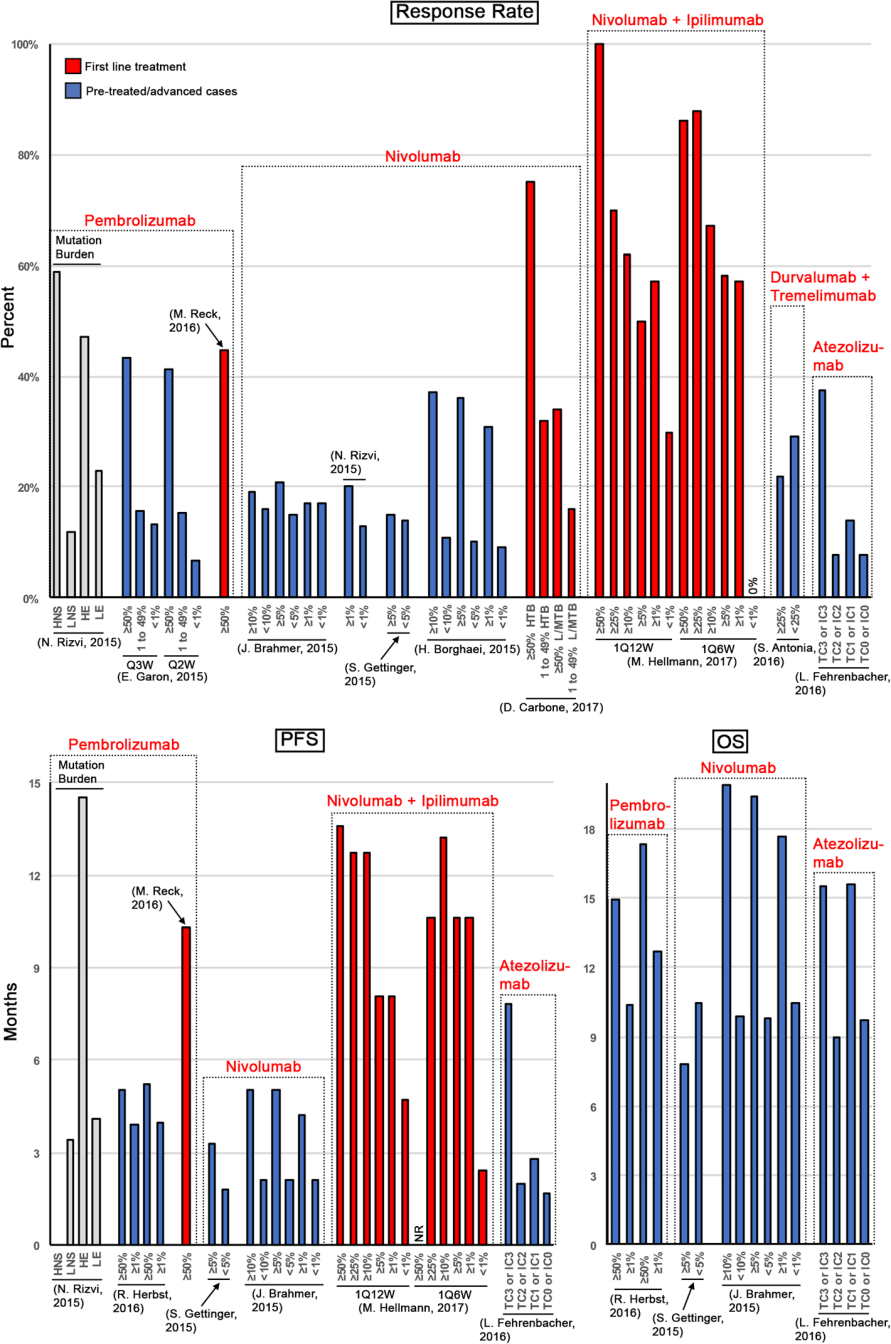
Consideration of PD-L1 and/or tumor-mutation burden in clinical trials of immune checkpoint inhibitors in non-small cell lung cancer. Shown are response rates, progression free survival (PFS) and overall survival (OS) based on PD-L1 expression levels and/or tumor-mutation burden for twelve published clinical trials (See Supplemental Materials for complete references). The x-axis indicates tumor mutation burden and/or percentage of PD-L1 positive cells (tumor cells unless otherwise indicated). HNS = high nonsynonymous # of mutations (median of 324), LNS = low nonsynonymous # of mutations (median of 122), HE = high exonic # of mutations (median of 494), LE = low exonic # of mutations (median of 190); Q3W = 10 mg/kg of pembrolizumab every 3-weeks, Q2W = 10 mg/kg of pembrolizumab every 2-weeks; TC0 (percent of PD-L1 positive tumor cells, < 1%) or IC0 (percent of PD-L1 positive tumor-infiltrating immune cells, < 1%), TC1 (≥1 and < 5%) or IC1 (≥1 and < 5%), TC2 (≥5 and < 50%) or IC2 (≥5 and < 10%), TC3 (≥50%) or IC3 (≥10%); 1Q12W = ipilimumab every12-weeks, 1Q6W = ipilimumab every 6-weeks; HTB = high tumor-mutational burden (≥243 somatic missense mutations), L/MTB = low/medium tumor-mutation burden (low = < 100 somatic missense mutations; medium = 100–242 somatic missense mutations)

Breakthrough studies have contributed to the approval of six major immune checkpoint inhibitors (ICIs): ipilimumab (anti-CTLA4), pembrolizumab (anti-PD-1), nivolumab (anit-PD-1), atezolizumab (anit-PD-L1), avelumab (anit-PD-L1) and durvalumab (anti-PD-L1) [7, 8]. Outcomes for these inhibitors vary widely and are dependent upon a number of factors. In NSCLC for cases where ≥50% of tumor cells express PD-L1, response rates as high as 44.8% have been reported [9]. Response rates can be significantly less robust if PD-L1 expression is not considered or is expressed at low levels (Fig. 1; Additional file 1: Table S1).

irAEs are generally mild and can be managed medically; however, severe adverse events, such as colonic perforation, a rare but life-threatening event that has been reported to occur in 1.0 to 1.5% of patients treated with ICIs, are predicted to continue to increase in incidence as immune inhibitors are used with increasing frequency and as patients are treated with multiple ICIs simultaneously [1, 10]. This case report highlights the importance of using molecular markers (Fig. 1; Additional file 1: Table S1) to determine the likelihood of treatment success and to limit irAEs. Furthermore, this monograph highlights that reinitiation of pembrolizumab treatment, even after immune-related bowel perforation, may be appropriate and that the current recommendation to permanently discontinue ICI treatment in the case of perforation should be considered on an individual basis.

## Case presentation

**Fig. 2:**
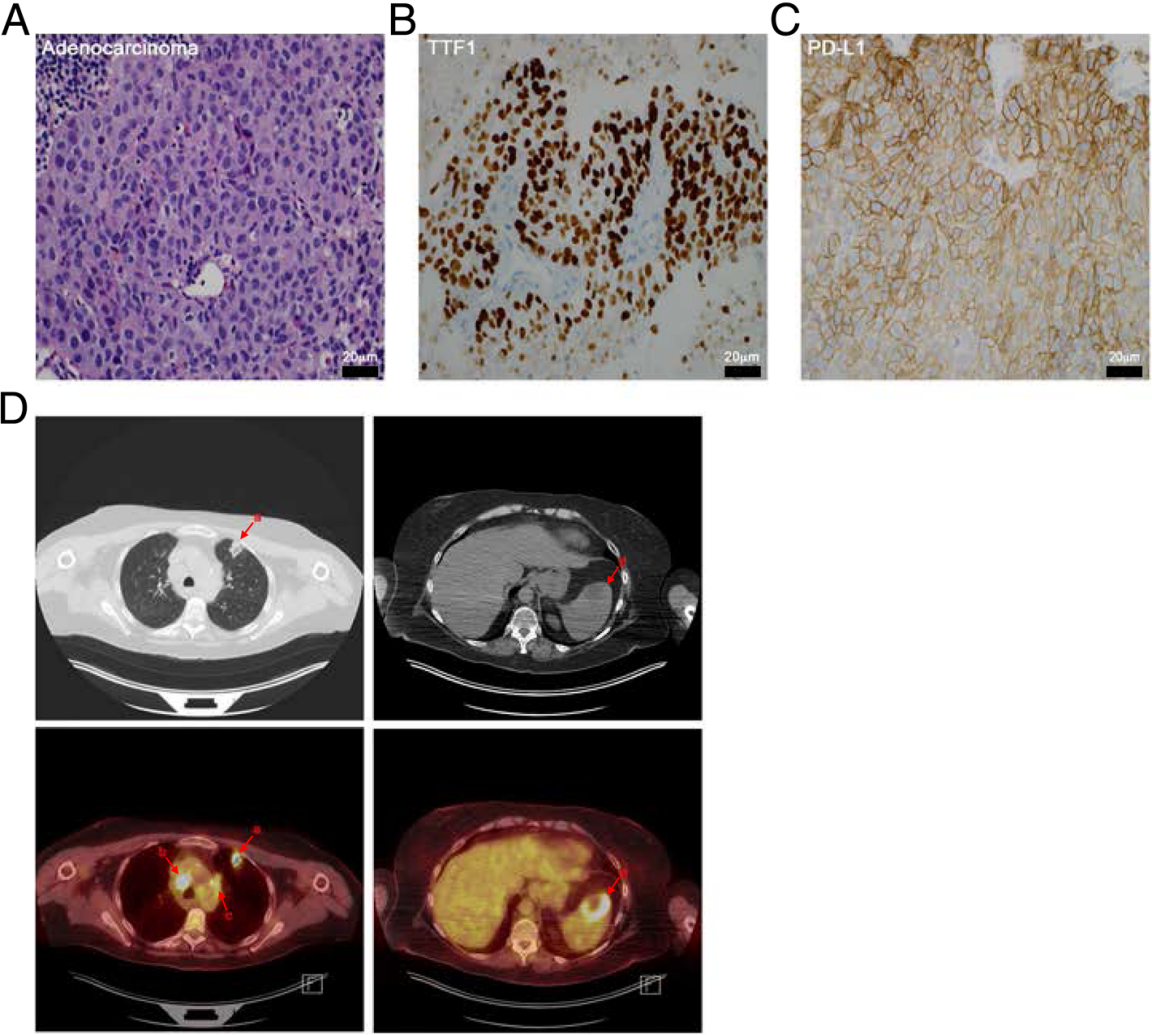
Diagnosis of PD-L1 positive, metastatic NSCLC and pre-treatment PET/CT imaging. **a**. Hematoxylin and eosin (H&E) stained neck mass biopsy showing adenocarcinoma cells consistent with primary lung disease. Scale bar = 20 mm. **b**. Thyroid transcription factor 1 (TTF-1) positive (dark brown staining) tumor cells. Bar = 20 mm. **c**. Immunohistochemical staining of the patient’s biopsy sample for PD-L1 (Dako 22C3). In the representative image, greater than 80% of tumor cells were PD-L1 positive (brown staining). Scale bar = 20 mm. **d**. Transverse computed tomography (CT) images with matching positron emission tomography (PET) images below. Detected lesions: *a* = anterior left upper lung lobe, *b* = right paratrachel region, *c* = left lung hilum, *d* = single splenic lesion

The patient in this case report (Additional file 1: Figure S1), is a 62-year-old woman with a 35-pack year smoking history, who presented with an enlarging, non-tender right neck mass, hoarseness and a twenty-pound weight loss. The initial differential included primary head and neck cancer versus metastatic disease. A subsequent neck biopsy reveled adenocarcinoma consistent with primary lung disease (Fig. 2a): found to be positive for thyroid transcription factor 1 (TTF-1) and negative for p40 and thyroglobulin (Fig. 2b).

Molecular studies of the patient’s biopsy were ordered. Wild type EGFR and no ALK or ROS1 rearrangements were detected, precluding the patient from targeted tyrosine kinase inhibitors. However, immunohistochemical (IHC) staining indicated that 80% of the patient’s tumor cells expressed PD-L1 (Fig. 2c), predicting a favorable response to immune checkpoint inhibition (Fig. 1; Additional file 1: Table S1) [3, 4, 11]. The patient was started on a three-week cycle of 200 mg pembrolizumab.

The primary mass on baseline staging was a 17 × 13 mm left upper lobe lesion consistent with primary lung cancer as well as multiple positron emission tomography (PET) avid lesions. PET/computed tomography (PET/CT) imaging for staging revealed multi-station mediastinal adenopathy, the right paratracheal region, the pre-carinal region, the right neck and the aortopulmonary window; left hilar adenopathy was also seen, and a single splenic lesion was also identified (Fig. 2d). The patient was therefore diagnosed with stage IV lung cancer (cT1aN3M1b).

**Fig. 3:**
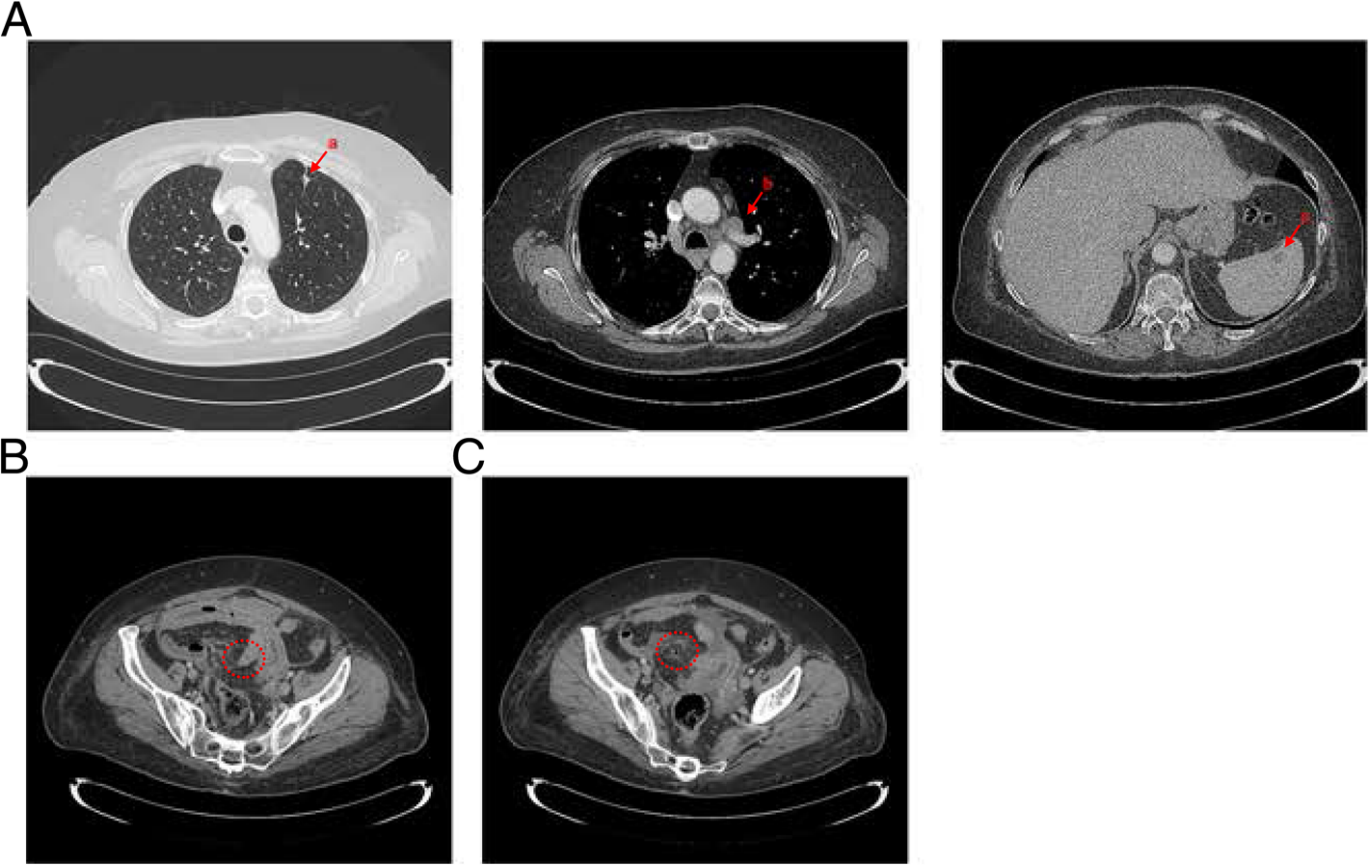
Post-treatment CT images of the lungs and spleen and abdominal images indicating small bowel perforation. **a**. Transverse CT images of the chest and abdomen showing significant reduction of the (*a*) anterior left upper lung lobe lesion, (*b*) left lung hilar lesion and (*c*) single splenic lesion. **b**. Transverse CT image showing submucosal edema (“target sign”) of the small bowel (red circle). **c**. Transverse CT image showing foci of non-dependent extraluminal air adjacent to the bowel (red circle)

The patient responded well to pembrolizumab and significant reduction in tumor burden was observed within ten weeks. Imaging showed reduction in size of the left upper lobe mass, the mediastinal lymphadenopathy and a reduction in the size of the splenic mass: collectively consistent with treatment effect for metastatic disease (Fig. 3a). Of note, after nine weeks of treatment, the patient’s thyroid function dropped precipitously, and the patient was diagnosed with hypothyroidism secondary to immunotherapy, necessitating levothyroxine treatment.

Regrettably, the patient experienced a second, more serious irAE, in the form enteritis, presenting in a clinically atypical form, without diarrhea. Constipation and abdominal discomfort developed around week eight of treatment. At that time, the patient presented to the direct referral unit at our center and imaging showed possible partial small bowel obstruction. The patient was hydrated and treated with metoclopramide; she declined an NG tube. Furthermore, she declined hospital admission. She progressively started to feel better for another four to five days after this discharge. An outpatient gastroenterology referral was placed, which the patient did not follow-up on.

The symptoms worsened significantly after the week ten-treatment cycle of pembrolizumab, forcing the patient to seek emergent medical care. The patient presented to the emergency department with anorexia, worsening continuous abdominal pain, nausea, vomiting and tachycardia, lasting for about ten days. On physical examination, the patient had involuntary guarding and rebound tenderness in the lower abdominal quadrants. Laboratory results indicated an elevated total white blood cell (WBC) count of 15.1. CT imaging with contrast of the abdomen and pelvis showed signs concerning for mural thickening of the proximal to mid jejunum, in the area of the mid pelvic cavity, with mucosal and submucosal edema and enhancement, concerning for a target sign and suggestive of ischemic etiology. Additionally, the patient had a cluster of mesenteric vessels concerning for mesenteric volvulus or internal hernia in the midline region of the pelvic cavity (Fig. 3b). Several small foci of non-dependent extraluminal air adjacent to the bowel and a trace amount of free fluid were detected (Fig. 3c).

**Fig. 4:**
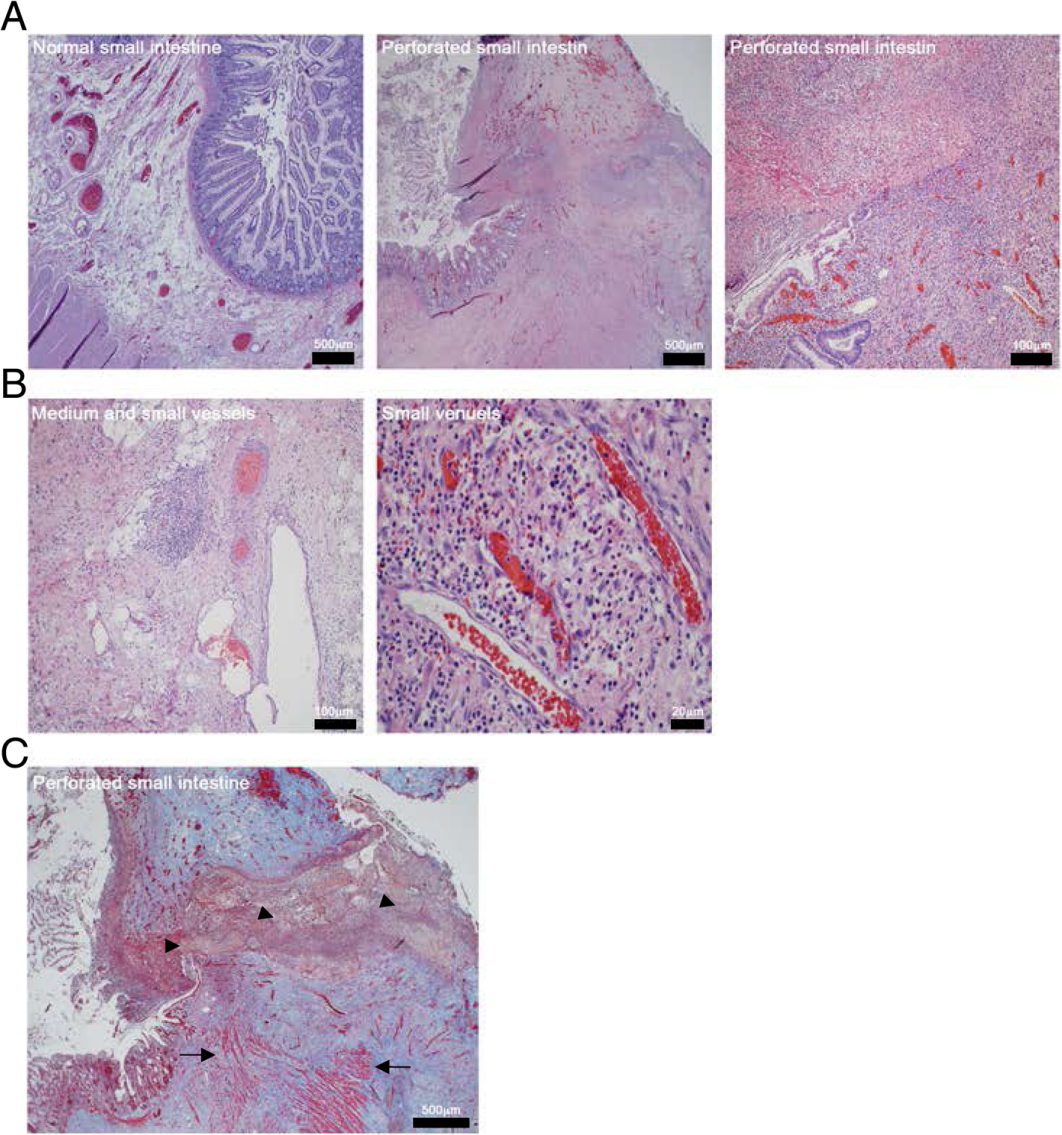
Microscopy evaluation of small bowel perforation. **a**. Hematoxylin and eosin (H&E) stained slides of normal small intestine (*left panel*), low magnification of perforated small intestine showing ulceration and perforation (*center panel*) and high magnification of perforated small intestine showing acute inflammation and mucosal ulceration (*right panel*). **b**. H&E slides of medium (*left panel*) and small (*left and right panel*) vessels associated with the small intestine. No evidence of vasculitis is seen. **c**. Section of small intestine H&E and trichrome stained showing ulceration with perforation through the serosa (arrow heads) with loss of muscularis propria (arrows) and acute and chronic inflammation and fibrosis

Exploratory laparotomy revealed one liter of purulent ascites. Part of the ileum was extremely erythematous and signs of perforation with significant inflammatory changes were evident. The concerning part of the small bowel was resected, and a primary anastomosis was created. The cecum, ascending colon, transverse, descending colon, sigmoid and rectum were without signs of injury. Surgical pathology of the resected portion of the small bowel showed focal, nonspecific, mesentery, non-caseating granulomatous inflammation, negative for tumor (Fig. 4a). Other commonly cited features were partially appreciated: there was indeed a lack of prominent intra-epithelial lymphocytes and crypt rupture; however, lamina propira expansion and villous blunting was not prominent [12].

Mesenteric vessels were negative for vasculitis and thromboembolism (Fig. 4b). Trichrome stain demonstrated loss of outer muscular wall due to ischemia and inflammation (Fig. 4c). Based on the pathological and surgical findings, the patient was diagnosed with pembrolizumab-associated small bowel perforation. Anti-TNF-α medications were not an appropriate treatment option due to fact that perforation of the bowel had occurred [13]. The patient recovered well from surgery. After extensive discussions, and with consideration of the patient’s remarkable response to treatment and the fact that she was resuming working full time and preferred to avoid chemotherapeutic side effects, the decision was made to resume pembrolizumab. Immunotherapy was restarted on post-operative day twenty-eight. Currently, twelve months since the start of treatment, the patient is continuing her immunotherapy with ongoing partial response and is able to continue her full-time job.

## Discussion and conclusions

### Response predictive markers

ICIs are revolutionizing the treatment of cancer, with an increasing number of patients benefiting from this class of therapeutic. To maximize treatment efficacy and to minimize severity of adverse events, establishing prognostic and response predictive markers is essential. Several response predictive markers have been identified (Fig. 1; Additional file 1: Table S1), two of which standout and one of which has been approved as standard pre-treatment assessment.

PD-L1 expression is now routinely assessed for cancer patients, with the recommended cutoff for anti-PD1 therapy being PD-L1 expression in ≥50% of tumor cells for frontline pembrolizumab treatment instead of chemotherapy [9], or PD-L1 expression in at least 1% of cells for second line treatment with pembrolizumab [14]. In NSCLC, the response rate to pembrolizumab has been reported to be 24.8% in previously untreated patients, with median progression free survival (PFS) and overall survival (OS) of 6.0 months and 16.2 months, respectively, when PD-L1 expression levels were not considered (Fig. 1; Additional file 1: Table S1). Among untreated patients with PD-L1 expression in ≥50% of tumor cells, the response rate, PFS and OS were significantly higher, with 50%, 12.5 months and not reached, respectively [4]. Garon et al. report that roughly 23.2% of patients with NSCLC have a PD-L1 score of at least 50% [4]. Importantly, PD-L1 expression has also been reported to frequently, 62% of the time, be concordant between primary tumor and metastases [15].

There remain several concerns regarding the detection and consideration of IHC detected PD-L1 expression: inconsistencies among PD-L1 specific antibodies; tumor heterogeneity in terms of PD-L1 expression; and dynamic changes in PD-L1 expression [11]. Additional studies are needed to help ensure clear and reliable standards when it comes to the assessment of PD-L1, particularly considering that the negative predictive value of PD-L1 IHC is not perfect.

A second particularly intriguing response predictive marker in lung cancer is based on overall mutational tumor load [16]. In advanced NSCLC, high nonsynonymous mutation burden (> 200) correlated with an increased rate of partial or stable responses to treatment with pembrolizumab, lasting at least 6 months. The response rate was 91% (10 of 11 patients) in cases with high mutational burden, compared to just 10% (1 of 10) in patients with low mutation burden (Fig. 1; Additional file 1: Table S1). Higher mutational burden also correlated with a significantly higher median progression free survival of 14.5 months compared to 3.7 months for cases with low mutational burden [17]. In the case of nivolumab treatment, patients with both, a high mutation burden and PD-L1 expression of ≥50% had a remarkably high response rate (75%), compared to 32 and 34% in patients with a high tumor-mutation burden or PD-L1 expression of < 50%, respectively [18, 19].

Several additional potential markers have been proposed as predictive for response to ICIs. For example, epithelial-mesenchymal transition (EMT) is known to play an important role in terms of therapy resistance in lung cancer [20] and there seems to be a correlation between EMT and resistance to immune checkpoint blockade as well [16]. T cell-inflammation gene expression profiles based on RNA levels – heavily focused on IFN-gamma-responsive genes, chemokine expression, cytotoxic activity and antigen presentation – showed promise in predicting PD-1 checkpoint blockade response across a wide variety of tumor types and is being developed as a clinical assay [21]. Lactate dehydrogenase (LDH) levels and absolute lymphocyte count have also been reported as promising [22]. In the case of nivolumab, a small prospective study has shown that patients with irAEs have improved progression free survival compared to patients who did not have irAEs: 6.4 months versus 1.5 months *P* = 0.01), respectively [23].

### ICI enterocolitis

Generally, enterocolitis presents with diarrhea, abdominal pain, nausea and vomiting. Several specific risk factors for ICI-associated enterocolitis have been identified and should be considered when ICI therapy is initiated. The type of treatment is relevant, with anti-CTLA-4 therapy carrying a higher risk of enterocolitis compared to anti-PD-1 therapy, and with combination therapy having the greatest probability of inducing colitis [24]. Not surprisingly, therapy dose is also important, with higher doses correlating with a higher risk of inducing therapy-associated colitis. Pre-treatment diagnosis of inflammatory bowel disease (IBD) is an additional risk factor for the development of enterocolitis. Microbiota enriched in *firmicutes* and poor in *Bacteroidetes* may also predispose patients to treatment-associated enterocolitis, specifically when treated with anti-CTLA-4 therapy [25]. Lastly, primary tumor histology appears to be significant, with melanoma being associated with a higher risk of treatment-associated enterocolitis compared to NSCLC and renal cell carcinoma (RCC) [24, 26, 27].

ICI-associated enterocolitis presents with distinguishing characteristics, depending on the specific molecular therapeutic target: CTLA-4 versus PD-1 [28]. Enteric biopsies after anti-CTLA-4 therapy have shown that CTLA-4-induced colitis presents with increased CD4+ T-cells within the lamina propria; whereas, PD-1-induced colitis generally presents with high mucosal and intraepithelial CD8+ T-cell populations. Furthermore, high mucosal TNF-α concentrations were only observed in cases of CTLA-4-induced colitis. Lower mucosal TNF-α levels correlated with steroid sensitivity [28].

Endoscopically, ICI-associated enterocolitis frequently presents with erythema, erosion, ulceration and luminal bleeding, which, as seen in this case report, can eventually lead to perforation [24]. Ulcerations have been reported in up to 79% of patients with enterocolitis and the majority of cases involve the distal colon. Endoscopic findings prior to the onset of symptoms do not however correlate with the occurrence of enterocolitis [29].

Anti-CTLA-4 induced enterocolitis frequently appears to be more severe compared to anti-PD-1 associated colitis, and shares some of the naturally occurring features associated with IBD: including both, Crohn’s disease and ulcerative colitis [30, 31]. The pathophysiology seems to involve development of antibodies to antigens of the enteric flora and thereby causing mucosal immunity dysregulation [29]. Nevertheless, histologically and in terms of fecal calprotectin levels and specific antibodies to enteric flora, ICI-induced colitis can be distinguished from IBD [29]. Additionally, both, elevated calprotectin prior to initiation of ICI treatment and rapid rise upon start of therapy were associated with severity of autoimmune-related colitis [13].

In terms of managing ICI-induced colitis, the main stay therapy is corticosteroids, which are effective against both, anti-CTLA-4 induced colitis and anti-PD-1 induced colitis. The majority of patients with colitis, 60–80%, respond to corticosteroids [24, 26]. Cases of steroid refractory colitis may require infliximab. Less well studied options include mesalazine, vedolizumab, tocilizumab and adalimumab-methotrexate [24]. Overall, TNF-α inhibitors are recommended for ICI-associated diarrhea, but should be avoided if the clinical picture is concerning for colonic perforation [25].

### Predictive irAE markers

The incidence rate of irAEs for ICIs has been reported to be 65% and higher [32]. This rate is likely to increase as dual ICI treatments are implement with increasing regularity [1]. Large clinical trials have reported minor adverse events such as rash, pruritus, fatigue, nausea and diarrhea to be most common; with more severe events, grade 3 and 4, including colitis, pneumonitis, neutropenia and colonic perforation occurring much less frequently [1, 32]. ICI associated colitis has been reported in 0.3 to 7% of patients treated with immune checkpoint inhibitors [33], with an estimated rate of 1.0 to 1.5% for colonic perforation [10]. The incidence of colitis can be substantially higher for combination treatment, having been reported to affect 56% of patients treated with nivolumab and ipilimumab [34–36].

Colitis caused by immune checkpoint inhibitors most commonly occurs within the rectum and sigmoid colon, but it can also affect the small intestine, as seen in this case report, and other regions of the upper gastrointestinal tract [10, 37]. In the case of ICI-associated colitis or enteritis, it is recommended to first rule out alternative causes, particularly infection with cytomegalovirus, hepatitis B virus and *Clostridium difficile*, celiac disease and inflammatory bowel disease [33]. Histologically, colitis associated with immune checkpoint inhibitors generally presents with acute focal or patchy areas of inflammation with infiltrating eosinophils and neutrophils and abscesses within the crypts [10]. Endoscopy with biopsies should be utilized to assess the regional extend of inflammation. Medical treatment includes corticosteroid treatment dosed at 1–2 mg/kg/day with a 1–2-month taper once symptoms have improved. In steroid refractory cases, treatment with infliximab (anti-TNF-α) is recommended [33].

Molecular markers for irAEs have not been investigated in as much detail as for therapeutic response rates; however, several potential candidates have emerged. These include immune related factors, such as elevated pre-treatment eosinophil levels; increased IL-17 (correlated with colitis); increased expression of CD177 and CEACAM1 (markers of neutrophil activation; associated with increased toxicity); increased neutrophil invasion and signs of inflammation on pre-treatment colonic biopsies (indicated higher incidence of digestive toxicity) [22]; and WBC count analysis [38]. Additional factors, including a family history of autoimmune disease, previous hepatitis or HIV infection and exposure to medication known to cause autoimmune toxicities, have been proposed as potential additional predictors of ICI-associated toxicity [39]. It has also been reported, based on a retrospective study of eighty-four patients treated with ipilimumab, that sarcopenia (OR = 5.34, 95% CI: 1.15–24.88, *P* = 0.033) and low muscle attenuation (defined as increased intramuscular adipose tissue; OR = 5.23, 95% CI: 1.41–19.30, *P* = 0.013) were significantly associated with high-grade adverse events [40].

## Conclusion

Our case highlights one of the most severe irAEs associated with immunotherapy and it lays out the management thereof as well as current and potential response and toxicity predictive markers. The enormous success of immune checkpoint inhibitors for the treatment of several different types of cancers continues to change the landscape of cancer treatment. To maximize the impact of these inhibitors, it is critical to systematically study treated patients and optimize treatment whilst minimizing undesirable outcomes. Further studies are needed to help better define predictive markers in terms of treatment response and probability of irAEs. This case also highlights that reinitiating pembrolizumab treatment after bowel perforation should be considered, albeit with caution, for specific patients.

## Additional file

Additional file 1: Supplemental Materials. **Table S1** Consideration of PD-L1 and/or tumor-mutation burden in clinical trials of immune checkpoint inhibitors in non-small cell lung cancer. **Figure S1.** Case timeline. (ZIP 74 kb)

## Abbreviations

ALK: Anaplastic lymphoma kinase
CD177: CD177 molecule
CEACAM1: Carcinoembryonic antigen related cell adhesion molecule 1
CI: Confidence interval
CT: Computed tomography
CTLA4: Cytotoxic T-lymphocyte associated protein 4
EGFR: Epidermal growth factor receptor
EMT: Epithelial-mesenchymal transition
HIV: Human immunodeficiency virus
ICI: Immune checkpoint inhibitor
IFN: Interferon
IHC: Immunohistochemical
IL-17: Interleukin 17
irAE: Immune-related adverse event
LDH: Lactate dehydrogenase
OS: Overall survival
PD-1: Proteins programmed cell death 1
PD-L1: Programmed cell death 1 ligand 1
PET: Positron emission tomography
PFS: Progression free survival
RNA: Ribonucleic acid
ROS1: ROS proto-oncogene 1
TNF-α: Tumor necrosis factor alpha
TTF-1: Thyroid transcription factor 1
WBC: White blood cell
