# Supplementary material for: Case report: reinitiating pembrolizumab treatment after small bowel perforation
Source: BMC Cancer. 2019 Apr 24;19:379. doi: 10.1186/s12885-019-5577-5 (PMC6482547; doi:10.1186/s12885-019-5577-5)
Supplement: Supplementary file 1 — Supplemental Materials. Table S1 Consideration of PD-L1 and/or tumor-mutation burden in clinical trials of immune checkpoint inhibitors in non-small cell lung cancer. Figure S1. Case timeline. (ZIP 74 kb) [file 12885_2019_5577_MOESM1_ESM.zip › Supplemental Materials1R5.docx]

**Case Report: Reinitiating Pembrolizumab Treatment after Small Bowel perforation**

Tim N. Beck, PhD, Alexander E. Kudinov, MD, Essel Dulaimi, MD and Yanis Boumber, MD, PhD

**Supplemental Materials**

**Supplement Table S1.** Consideration of PD-L1 and/or tumor-mutation burden in clinical trials of immune checkpoint inhibitors in non-small cell lung cancer.

**Supplemental Figure 1.** Case timeline.

**Supplemental References**

1. Rizvi NA, Hellmann MD, Snyder A, et al. Cancer immunology. Mutational landscape determines sensitivity to PD-1 blockade in non-small cell lung cancer. Science. 2015;348(6230):124-128.

2. Herbst RS, Baas P, Kim DW, et al. Pembrolizumab versus docetaxel for previously treated, PD-L1-positive, advanced non-small-cell lung cancer (KEYNOTE-010): a randomised controlled trial. Lancet. 2016;387(10027):1540-1550.

3. Garon EB, Rizvi NA, Hui R, et al. Pembrolizumab for the treatment of non-small-cell lung cancer. N Engl J Med. 2015;372(21):2018-2028.

4. Reck M, Rodriguez-Abreu D, Robinson AG, et al. Pembrolizumab versus Chemotherapy for PD-L1-Positive Non-Small-Cell Lung Cancer. N Engl J Med. 2016;375(19):1823-1833.

5. Brahmer J, Reckamp KL, Baas P, et al. Nivolumab versus Docetaxel in Advanced Squamous-Cell Non-Small-Cell Lung Cancer. N Engl J Med. 2015;373(2):123-135.

6. Rizvi NA, Mazieres J, Planchard D, et al. Activity and safety of nivolumab, an anti-PD-1 immune checkpoint inhibitor, for patients with advanced, refractory squamous non-small-cell lung cancer (CheckMate 063): a phase 2, single-arm trial. Lancet Oncol. 2015;16(3):257-265.

7. Gettinger SN, Horn L, Gandhi L, et al. Overall Survival and Long-Term Safety of Nivolumab (Anti–Programmed Death 1 Antibody, BMS-936558, ONO-4538) in Patients With Previously Treated Advanced Non–Small-Cell Lung Cancer. Journal of Clinical Oncology. 2015;33(18):2004-2012.

8. Borghaei H, Paz-Ares L, Horn L, et al. Nivolumab versus Docetaxel in Advanced Nonsquamous Non-Small-Cell Lung Cancer. N Engl J Med. 2015;373(17):1627-1639.

9. Carbone DP, Reck M, Paz-Ares L, et al. First-Line Nivolumab in Stage IV or Recurrent Non-Small-Cell Lung Cancer. N Engl J Med. 2017;376(25):2415-2426.

10. Hellmann MD, Rizvi NA, Goldman JW, et al. Nivolumab plus ipilimumab as first-line treatment for advanced non-small-cell lung cancer (CheckMate 012): results of an open-label, phase 1, multicohort study. The Lancet Oncology. 2017;18(1):31-41.

11. Antonia S, Goldberg SB, Balmanoukian A, et al. Safety and antitumour activity of durvalumab plus tremelimumab in non-small cell lung cancer: a multicentre, phase 1b study. Lancet Oncol. 2016;17(3):299-308.

12. Fehrenbacher L, Spira A, Ballinger M, et al. Atezolizumab versus docetaxel for patients with previously treated non-small-cell lung cancer (POPLAR): a multicentre, open-label, phase 2 randomised controlled trial. Lancet. 2016;387(10030):1837-1846.
