# Supplementary material for: Case report: reinitiating pembrolizumab treatment after small bowel perforation
Source: BMC Cancer. 2019 Apr 24;19:379. doi: 10.1186/s12885-019-5577-5 (PMC6482547; doi:10.1186/s12885-019-5577-5)
Supplement: Supplementary file 1 — Supplemental Materials. Table S1 Consideration of PD-L1 and/or tumor-mutation burden in clinical trials of immune checkpoint inhibitors in non-small cell lung cancer. Figure S1. Case timeline. (ZIP 74 kb) [file 12885_2019_5577_MOESM1_ESM.zip › Supplemental Table S1 copyR5.docx]

| **Supplemental Table S1** | |  |  |  |  |  |
| --- | --- | --- | --- | --- | --- | --- |
|  | |  |  |  |  |  |
| **Mutational Burden** | |  |  |  |  |  |
|  | |  |  |  |  |  |
| 2015 | |  |  |  |  |  |
| Naiyer A. Rizvi, et al. | |  |  |  |  |  |
| http://science.sciencemag.org/content/348/6230/124.full | |  |  |  |  |  |
| Mutational landscape determines sensitivity to PD-1 blockade in non–small cell lung cancer | | |  |  |  |  |
|  | |  |  |  |  |  |
| **Patient types:** | | stage IV |  |  |  |  |
| **Treatment:** | | pembrolizumab (every 2-3 weeks) |  |  |  |  |
|  | | **Patient number (n)** | **ORR** | **PFS** |  |  |
| **High nonsynonymous (median 324)** | | 17 | 59% | NR |  |  |
| **Low nonsynonymous (median 122)** | | 17 | 12% | 3.4 |  |  |
| **High exonic (median 494)** | | 17 | 47% | 14.5 |  |  |
| **Low exonic (median 190)** | | 17 | 23% | 4.1 |  |  |
|  | |  |  |  |  |  |
|  | |  |  |  |  |  |
| **PD-L1** | |  |  |  |  |  |
|  | |  |  |  |  |  |
| 2016 | |  |  |  |  |  |
| Prof Roy S Herbst, et al. | |  |  |  |  |  |
| http://www.thelancet.com/journals/lancet/article/PIIS0140-6736(15)01281-7/fulltext | | |  |  |  |  |
| Pembrolizumab versus docetaxel for previously treated, PD-L1-positive, advanced non-small-cell lung cancer  (KEYNOTE-010): a randomised controlled trial | | | | | | |
| **Patient types:** | | Pre-treated with progression (plantinum doublet as well as TKI for EGFR/ALK) |  |  |  |  |
| **Treatment:** | | pembrolizumab (every 3 weeks) | **2mg/kg** | **10mg/kg** | **2mg/kg** | **10mg/kg** |
|  | | **Patient number (n)** | **OS** | **OS** | **PFS** | **PFS** |
| **All patients (>=1%)** | | 344 | 10.4 |  | 3.9 |  |
| **All patients (>=1%)** | | 346 |  | 12.7 |  | 4 |
| **>=50%** | | 139 | 14.9 |  | 5 |  |
| **>=50%** | | 151 |  | 17.3 |  | 5.2 |
|  | |  |  |  |  |  |
| 2015 | |  |  |  |  |  |
| http://www.nejm.org/doi/full/10.1056/NEJMoa1501824#t=article | |  |  |  |  |  |
| Edward B. Garon, et al. | |  |  |  |  |  |
| Pembrolizumab for the Treatment of Non–Small-Cell Lung Cancer | |  |  |  |  |  |
| **Patient types:** | | locally advanced or metastatic non–small-cell lung cancer,ECOG performance status of 1 or less |  |  |  |  |
| **Treatment:** | | pembrolizumab | 10mg/kg Q3W | 10mg/kg Q2W |  |  |
|  | | **Patient number (n)** | **ORR** | **ORR** |  |  |
| **>=50%** | | 44 | 43.20% |  |  |  |
| **>=50%** | | 34 |  | 41.20% |  |  |
| **1 to 49%** | | 57 | 15.80% |  |  |  |
| **1 to 49%** | | 55 |  | 15.40% |  |  |
| **<1%** | | 15 | 13.30% |  |  |  |
| **<1%** | | 15 |  | 6.70% |  |  |
|  | |  |  |  |  |  |
| 2016 | |  |  |  |  |  |
| http://www.nejm.org/doi/full/10.1056/NEJMoa1606774#t=article | |  |  |  |  |  |
| Martin Reck, et al. | |  |  |  |  |  |
| Pembrolizumab versus Chemotherapy for PD-L1–Positive Non–Small-Cell Lung Cancer | | |  |  |  |  |
| **Patient types:** | | first-line therapy |  |  |  |  |
| **Treatment:** | | Pembrolizumab |  |  |  |  |
|  | | **Patient number (n)** | **ORR** | **PFS** |  |  |
| **>=50%** | | 154 | 44.80% | 10.3 |  |  |
|  | |  |  |  |  |  |
| 2015 | |  |  |  |  |  |
| http://www.nejm.org/doi/10.1056/NEJMoa1504627#t=articleTop | |  |  |  |  |  |
| Julie Brahmer, et al. | |  |  |  |  |  |
| Nivolumab versus Docetaxel in Advanced Squamous-Cell Non–Small-Cell Lung Cancer | | |  |  |  |  |
| **Patient types:** | | disease progressed on platinum therapy |  |  |  |  |
| **Treatment:** | | nivolumab |  |  |  |  |
|  | | **Patient number (n)** | **ORR** |  |  |  |
| **>= 1% Niv** | | 63 | 17% |  |  |  |
| **<1% Niv** | | 54 | 17% |  |  |  |
| **>= 5% Niv** | | 42 | 21% |  |  |  |
| **<5% Niv** | | 75 | 15% |  |  |  |
| **>= 10% Niv** | | 36 | 19% |  |  |  |
| **<10% Niv** | | 81 | 16% |  |  |  |
|  | |  |  |  |  |  |
| 2015 | |  |  |  |  |  |
| http://www.thelancet.com/journals/lanonc/article/PIIS1470-2045(15)70054-9/fulltext | | |  |  |  |  |
| Dr Naiyer A Rizvi, et al. | |  |  |  |  |  |
| Activity and safety of nivolumab, an anti-PD-1 immune checkpoint inhibitor, for patients with advanced, refractory  squamous non-small-cell lung cancer (CheckMate 063): a phase 2, single-arm trial | | | | | | |
| **Patient types:** | | pre-treated with progression or recurrence |  |  |  |  |
| **Treatment:** | | nivolumab (every 2 weeks) |  |  |  |  |
|  | | **Patient number (n)** | **PR** |  |  |  |
| **>= 1%** | | 45 | 20% |  |  |  |
| **<1%** | | 31 | 13% |  |  |  |
|  | |  |  |  |  |  |
| 2015 | |  |  |  |  |  |
| http://ascopubs.org/doi/full/10.1200/JCO.2014.58.3708 | |  |  |  |  |  |
| Scott N. Gettinger, et al. | |  |  |  |  |  |
| Overall Survival and Long-Term Safety of Nivolumab (Anti–Programmed Death 1 Antibody, BMS-936558, ONO-4538) in  Patients With Previously Treated Advanced Non–Small-Cell Lung Cancer | | | | | | |
| **Patient types:** | | previously treated |  |  |  |  |
| **Treatment:** | | nivolumab (every 2 weeks) |  |  |  |  |
|  | | **Patient number (n)** | **ORR** | **PFS** | **OS** |  |
| **>=5%** | | 33 | 15% | 3.3 | 7.8 |  |
| **<5%** | | 35 | 14% | 1.8 | 10.5 |  |
|  | |  |  |  |  |  |
| 2015 | |  |  |  |  |  |
| http://www.nejm.org/doi/full/10.1056/NEJMoa1507643#t=article | |  |  |  |  |  |
| Hossein Borghaei, et al. | |  |  |  |  |  |
| Nivolumab versus Docetaxel in Advanced Nonsquamous Non–Small-Cell Lung Cancer | | |  |  |  |  |
| **Patient types:** | | previously treated patients |  |  |  |  |
| **Treatment:** | | nivolumab |  |  |  |  |
|  | | **Patient number (n)** | **ORR** | **PFS** | **OS** |  |
| **>= 1% Niv** | | 123 | 31% | 4.2 | 17.7 |  |
| **<1% Niv** | | 108 | 9% | 2.1 | 10.5 |  |
| **>= 5% Niv** | | 95 | 36% | 5 | 19.4 |  |
| **<5% Niv** | | 136 | 10% | 2.1 | 9.8 |  |
| **>= 10% Niv** | | 86 | 37% | 5 | 19.9 |  |
| **<10% Niv** | | 145 | 11% | 2.1 | 9.9 |  |
|  | |  |  |  |  |  |
| 2017 | |  |  |  |  |  |
| http://www.sciencedirect.com/science/article/pii/S1470204516306246?via%3Dihub | | |  |  |  |  |
| Matthew D. Hellmann, et al. | |  |  |  |  |  |
| Nivolumab plus ipilimumab as first-line treatment for advanced non-small-cell lung cancer (CheckMate 012): results of an  open-label, phase 1, multicohort study | | | | | | |
| **Patient types:** | | first line |  |  |  |  |
| **Treatment:** | | nivolumab plus ipilimumab |  |  |  |  |
|  | | **Patient number (n)** | **ORR** | **PFS** |  |  |
| **<1% 3Q2W + 1 Q12W** | | 10 | 30% | 4.7 |  |  |
| **>=1%** | | 21 | 57% | 8.1 |  |  |
| **>=5%** | | 16 | 50% | 8.1 |  |  |
| **>=10%** | | 13 | 62% | 12.7 |  |  |
| **>=25%** | | 10 | 70% | 12.7 |  |  |
| **>=50%** | | 6 | 100% | 13.6 |  |  |
| **<1% 3Q2W + 1 Q6W** | | 7 | 0% | 2.4 |  |  |
| **>=1%** | | 23 | 57% | 10.6 |  |  |
| **>=5%** | | 18 | 58% | 10.6 |  |  |
| **>=10%** | | 15 | 67% | 13.2 |  |  |
| **>=25%** | | 8 | 88% | 10.6 |  |  |
| **>=50%** | | 7 | 86% | NR |  |  |
|  | |  |  |  |  |  |
| 2016 | |  |  |  |  |  |
| http://www.sciencedirect.com/science/article/pii/S1470204515005446 | |  |  |  |  |  |
| Prof Scott Antonia, et al. | |  |  |  |  |  |
| Safety and antitumour activity of durvalumab plus tremelimumab in non-small cell lung cancer: a multicentre, phase 1b study | | | | |  |  |
| **Patient types:** | | pre-treated but immunotherapy naïve |  |  |  |  |
| **Treatment:** | | durvalumab plus tremelimumab |  |  |  |  |
|  | | **Patient number (n)** | **ORR** |  |  |  |
| **>=25%** | | 9 | 22% |  |  |  |
| **<25%** | | 14 | 29% |  |  |  |
|  | |  |  |  |  |  |
| 2016 | |  |  |  |  |  |
| http://www.thelancet.com/journals/lancet/article/PIIS0140-6736(16)00587-0/fulltext | | |  |  |  |  |
| Louis Fehrenbacher, et al. | |  |  |  |  |  |
| Atezolizumab versus docetaxel for patients with previously treated non-small-cell lung cancer (POPLAR): a multicentre,  open-label, phase 2 randomised controlled trial | | | | | | |
| **Patient types:** | Previously treated | |  |  |  |  |
| **Treatment:** | atezolizumab (every 3 weeks) | |  |  |  |  |
|  | **Patient number (n)** | | **ORR** | **PFS** | **OS** |  |
| **TC3 (>=50%) or IC3 (>=10%)** | 47 | | 37.50% | 7.8 | 15.5 |  |
| **TC2 (>=5 <50%) or IC2 (>=5 <10)** | 58 | | 7.70% | 2 | 9 |  |
| **TC1 (>=1 <5%) or IC1 (>=1 <5%)** | 90 | | 14.00% | 2.8 | 15.6 |  |
| **TC0 (<1%) or IC0 (<1%)** | 92 | | 7.80% | 1.7 | 9.7 |  |
|  |  | |  |  |  |  |
| **PD-L1 and tumor mutation burden** |  | |  |  |  |  |
|  |  | |  |  |  |  |
| 2017 |  | |  |  |  |  |
| http://www.nejm.org/doi/full/10.1056/NEJMoa1613493#t=article |  | |  |  |  |  |
| David P. Carbone, et al. |  | |  |  |  |  |
| First-Line Nivolumab in Stage IV or Recurrent Non–Small-Cell Lung Cancer |  | |  |  |  |  |
| **Patient types:** | first line | |  |  |  |  |
| **Treatment:** | nivolumab | |  |  |  |  |
|  | **Patient number (n)** | | **ORR** |  |  |  |
| **>=50% and High tumor burden (>= 243)** | 16 | | 75% |  |  |  |
| **1-49% and High tumor burden (>= 243)** | 41 | | 32% |  |  |  |
| **>=50% and Low/Med tumor burden (<243)** | 41 | | 34% |  |  |  |
| **1-49% and Low/Med tumor burden (<243)** | 70 | | 16% |  |  |  |
