# Supplementary figures and images for: Case report: reinitiating pembrolizumab treatment after small bowel perforation
Source: BMC Cancer. 2019 Apr 24;19:379. doi: 10.1186/s12885-019-5577-5 (PMC6482547; doi:10.1186/s12885-019-5577-5)

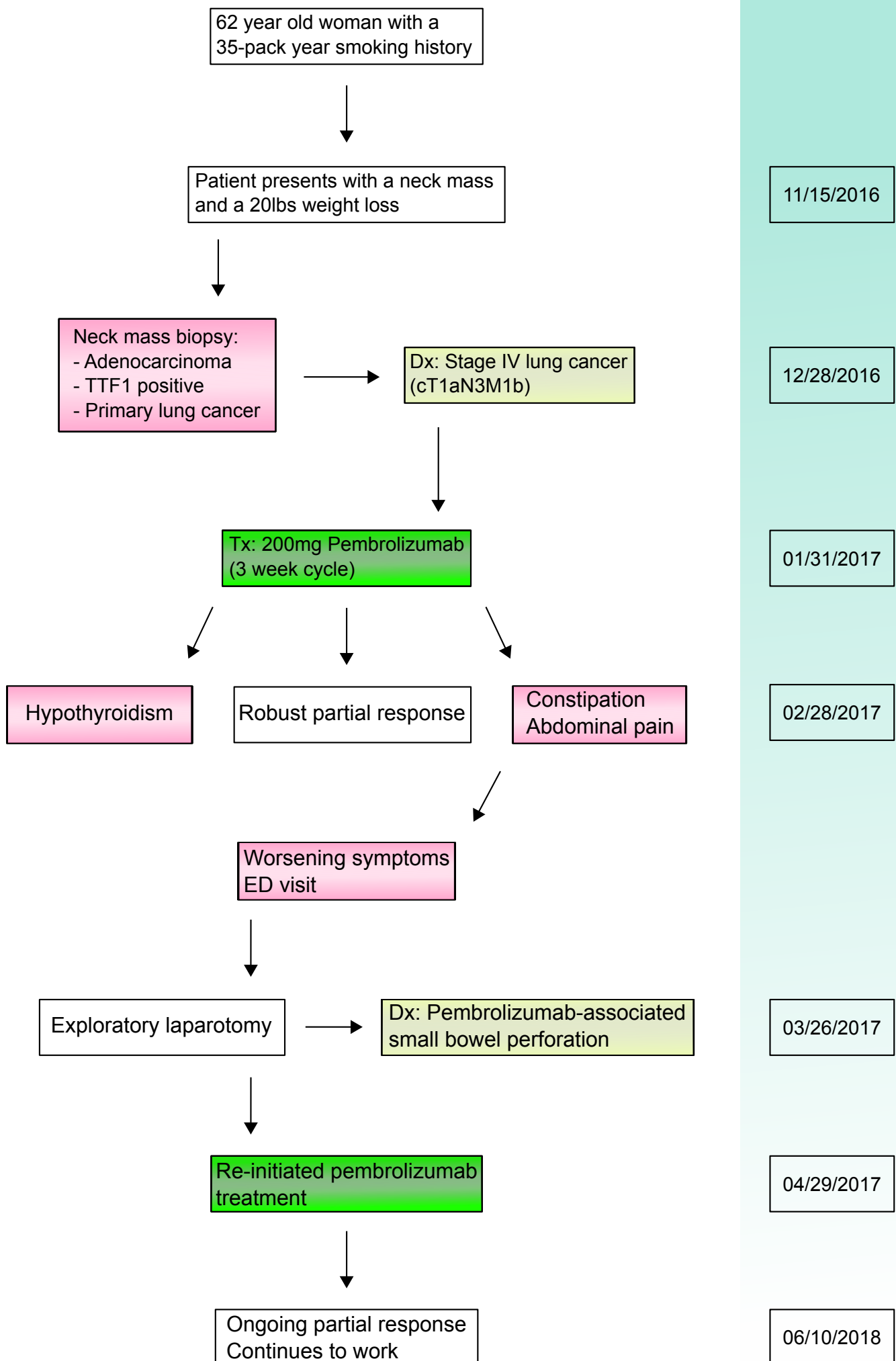

Supplement: Supplementary file 1 — Supplemental Materials. Table S1 Consideration of PD-L1 and/or tumor-mutation burden in clinical trials of immune checkpoint inhibitors in non-small cell lung cancer. Figure S1. Case timeline. (ZIP 74 kb) [file 12885_2019_5577_MOESM1_ESM.zip › Supplemental_Fig1R5.pdf]
